# Diagnostic accuracy of positron emission tomography/computerized tomography for periprosthetic joint infection of hip: systematic review and meta-analysis

**DOI:** 10.1186/s13018-023-04061-4

**Published:** 2023-08-30

**Authors:** Hongning Hua, Jinwen Liu

**Affiliations:** 1 Shanxi Bethune Hospital, Shanxi Academy of Medical Sciences, Tongji Shanxi Hospital, Third Hospital of Shanxi Medical University, Taiyuan, 030032 China; 2 Tongji Hospital, Tongji Medical College, Huazhong University of Science and Technology, Wuhan, 430030 China

**Keywords:** Diagnostic accuracy, Meta-analysis, Periprosthetic joint infection, Positron emission tomography

## Abstract

**Background:**

American Academy of Orthopaedic Surgeons (AAOS) has provided the guidelines for diagnosing a patient with periprosthetic joint infection including the use of positron emission tomography/computed tomography (PET/CT). Systematic evidence focussing on periprosthetic joint infection (PJI) of hip is limited, which also contains limited number of studies. Hence, the current study aims to perform a pooled analysis of all studies that have assessed the diagnostic accuracy of PET/CT for PJI of hip.

**Methods:**

Searches were done in PubMed Central, EMBASE, MEDLINE, SCOPUS and Cochrane library until December 2022. Meta-analysis was carried out using random-effects model. With 95% confidence intervals (CIs), pooled sensitivity and specificity were reported.

**Results:**

Twenty-six studies met the inclusion criteria. The pooled sensitivity of PET/CT was 89% (95% CI 84–93%), while the pooled specificity was 86% (95% CI 79–91%). The AUROC was 0.94 (95% CI 0.72–0.99). There was statistically significant heterogeneity (*p* < 0.001) with I2 value of 96%. The diagnostic odds ratio was 52 (95% CI 26–106). Likelihood ratio positive was 6.5 (95% CI 4.1–10.3) and negative was 0.13 (95% CI 0.08–0.19).

**Conclusion:**

Our study found that PET/CT was found to have higher level of accuracy in terms of sensitivity and specificity. Further large-scale research can help to find answers for such questions and provide final conclusive evidence on the inclusion of the imaging modality into the routine clinical practice guidelines for suspected periprosthetic joint infection patients.

**Supplementary Information:**

The online version contains supplementary material available at 10.1186/s13018-023-04061-4.

## Introduction

A large number of patients worldwide have hip prosthetics. The prevalence of patients with any prosthesis is nearly 3% amongst middle aged and older adults and it seems to double by 80 years [1]. With an ageing population, the number of hip joint arthroplasty procedures has risen in recent decades. By 2060, the number of hip arthroplasty procedures is expected to rise by nearly 40% [2]. However, there is a limitation in that a significant portion of these prosthetics require revision, with the rate rising to nearly 15% after ten years [3]. The commonest cause of this revision is mainly aseptic or septic loosening, which contribute to about two-third of total revisions followed by dislocations and fractures [4]. This shows the importance of differentiating the aseptic and septic loosening, which is necessary to plan the treatment for the patients [5]. However, differentiating these conditions can be difficult with the existing clinical procedures.

The American Academy of Orthopaedic Surgeons (AAOS) has provided the guidelines for diagnosing a patient with periprosthetic joint infection, which recommends testing such as erythrocyte sedimentation rate (ESR) and C-reactive protein (CRP), as well as the use of positron emission tomography/computed tomography (PET/CT) in certain cases [6]. Amongst these procedures, role of PET-CT is particularly important, given its higher level of accuracy compared to rest of the parameters.7 PET-CT also has several advantages in terms of convenience to the patients, no requirement for the cell labelling (unlike white blood cell scintigraphy), and the entire diagnostic procedure takes not more than two hours [7].

Though several primary studies and fewer secondary meta-analyses are available on this topic [8–10], systematic evidence focussing on periprosthetic joint infection of hip is limited, which also contains limited number of studies. Specifically focussing on periprosthetic joint infection of hip will provide guidance and frame guidelines for specific procedure and site of infection. Hence, the current study aims to search for and perform a pooled analysis of all studies that have assessed the diagnostic accuracy of PET/CT for periprosthetic joint infection.

### Objective

The objective of the present investigation is to conduct a comprehensive search and meta-analysis of all relevant studies that have evaluated the diagnostic precision of PET/CT in the context of periprosthetic joint infection.

## Materials and methods

### Inclusion criteria

#### Type of studies

We included research articles that looked at how well PET/CT works for detecting periprosthetic joint infection at hip, regardless of the design of the study, contrast material used. We included studies that were published as full text or grey literature (i.e. unpublished data) in the form of thesis, conference abstracts, etc. We did not include case reports, or case series or traditional review articles.

#### Index test

Studies utilizing PET/CT as the index test for diagnosing the periprosthetic joint infection of the hip joint.

## Reference standards

We only included studies that compared the accuracy of PET/CT for detecting periprosthetic joint infection with an intraoperative or postoperative confirmation of infection using standard microbiological/histopathological/clinical procedures.

### Outcome measure

We only included studies that reported the sensitivity and specificity of this diagnostic method, or that provided information that could be used to calculate these rates (like true positives, true negatives, false negatives and false positives).

### Search strategy

A thorough and methodical search of multiple electronic databases such as PubMed Central, EMBASE, MEDLINE, SCOPUS and Cochrane library were conducted. The keywords and search terms were determined during the planning stage. Both medical subject headings and free-text words were utilized to search the databases, and truncations, wildcards, and proximity searching were employed with the keywords and their synonyms. The same terms were also used to search for published studies in the Cochrane library, Scopus, and Embase. The search also included key concepts and corresponding subject headings in each database. The final search was a combination of the individual search results using appropriate Boolean operators. The search was limited to studies published in English and from the inception of the databases up to December 2022. Detailed search strategy is available in Additional file 1.

### Study selection

Two authors were responsible for the initial screening process, which involved evaluating the titles and abstracts of the literature search. Citations, along with their titles and abstracts, were added to a designated endnote library and duplicates were removed to create a final list of studies to be reviewed. The full text of these studies was then retrieved and evaluated by the same two authors against the inclusion criteria of the study. Studies that did not meet the criteria were excluded and the reasons for exclusion were recorded. The screening and selection process was illustrated using a PRISMA flow chart [11].

### Data extraction

Using a pre-designed data extraction form, information was gathered from the studies, including details such as the study design, setting, index test, reference standards, type of contrast agent used in PET/CT, sample size, average age, inclusion and exclusion criteria, test results and negatives. The data were then entered into STATA software and a third investigator reviewed the entered data to ensure accuracy against the original study reports.

### Risk of bias assessment

Two independent authors have assessed the risk of bias in the studies by using the "Quality Assessment of Diagnostic Accuracy Studies-2 (QUADAS-2) tool" [12]. The following areas were evaluated: patient selection, index tests, reference standards, and the flow and timing of assessments. The authors assigned a grade of high, low, or unclear for the potential source of bias in each of the included studies.

### Statistical analysis

To get the pooled values of sensitivity, specificity, likelihood ratio positive, negative, and summary diagnostic odds ratio for PET/CT, a meta-analysis was carried out using STATA 14.2 ("StataCorp, College Station, TX, USA"). It was used to create "Summary Receiver Operator Characteristic curves (sROC)" and the outcome was reported as area under ROC (AUROC). A forest plot was used to graphically display study-specific and combined estimations. Chi square test for heterogeneity, and I2 statistics to measure the inconsistency was performed. Publication bias was evaluated, graphically displayed, and tested using Deek's test for asymmetry in the funnel plot. Meta-regression was performed with the factors such as contrast agent, study design, study region, sample size and risk of bias assessment.

## Results

### Study selection

**Fig. 1:**
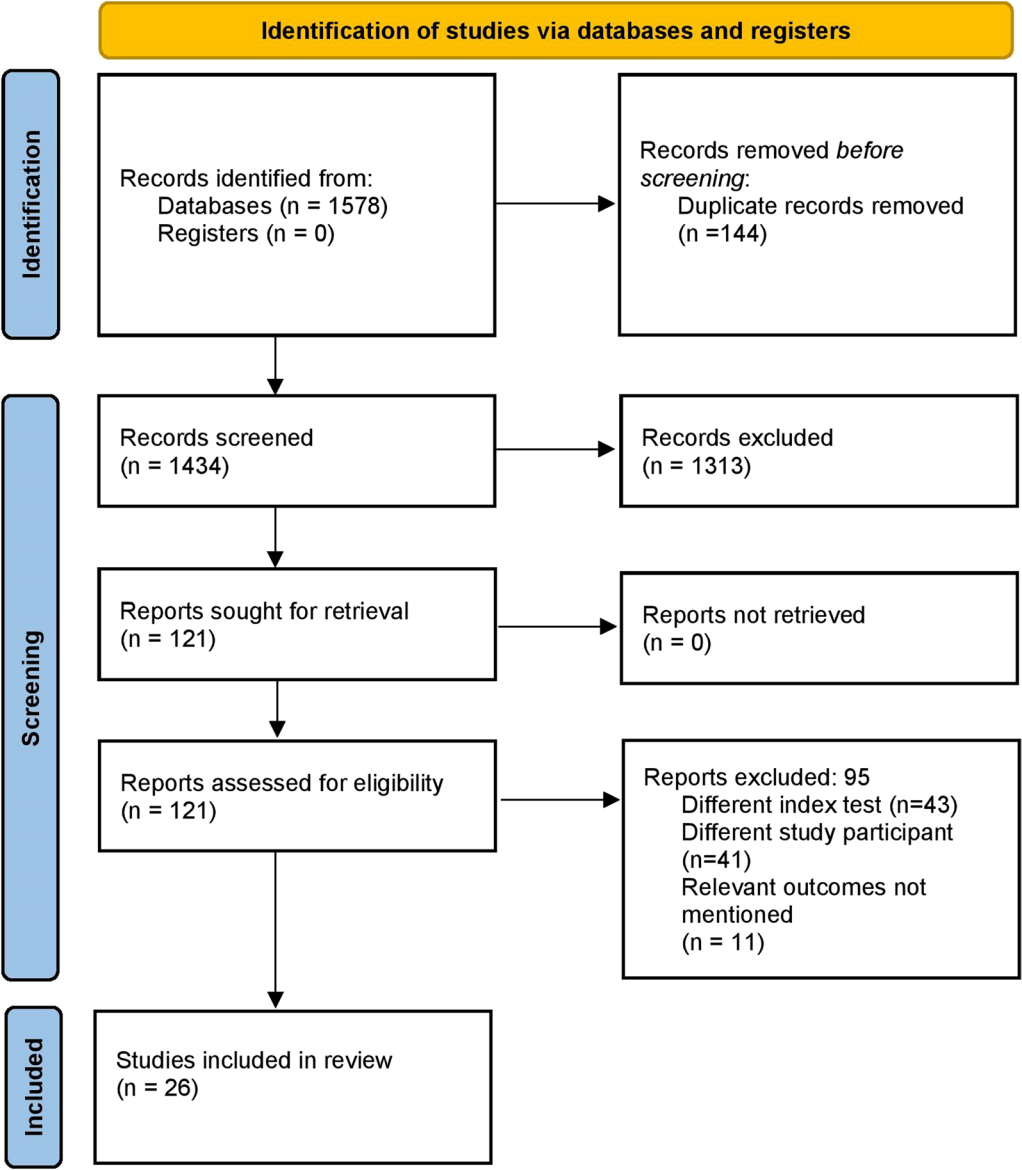
PRISMA flow chart

The systematic literature search yielded 1578 records, and 121 of those studies were determined to be pertinent for full-text retrieval. Four publications were found by manually examining the bibliographies of the retrieved studies. Finally, 26 studies satisfied all the inclusion criteria during the second round of screening and were incorporated in the analysis (Fig. 1) [8–10, 13–35].

### Characteristics of included studies

Table 1 shows the characteristics of the included studies. Majority (22 studies) were prospective studies. Most studies were done in USA (6 studies), Germany (4 studies), China (2 studies), The Netherlands (2 studies), Taiwan (2 studies) and India (2). Histopathological, microbiological and clinical findings were most commonly used as reference standard amongst the included studies. The sample size ranged from 10 to 134. Majority studies (21 studies) had lower risk of bias (Table 1).

**Table 1:** Characteristics of the included studies (*N* = 26)

Serial no	References	Country	Prospective/retrospective	Total sample	Contrast agent used for PET/CT	Gold standard for diagnosis	Mean age (in years)	Risk of bias
1	Aksoy et al. [24]	Turkey	Prospective	16	FDG	Postoperative histopathological/microbiological/clinical work-up	61	Low risk
2	Aleksyniene et al. [31]	Denmark	Prospective	25	FDG	Intraoperative findings and microbiological culture results and the clinical follow-up	Not reported	Low risk
3	Basu et al. [8]	USA	Prospective	134	FDG	Microbiological confirmation using cultures/purulent fluid and presence of neutrophilic infiltrate	57	Low risk
4	Chacko et al. [29]	USA	Prospective	41	FDG	Microbiology, histopathology, surgical and clinical follow-up	61.9	Low risk
5	Chen et al. [16]	Taiwan	Prospective	24	FDG	Intraoperative tissue cultures, intraoperative pathology, and clinical follow-up	Not reported	Low risk
6	Chryssikos et al. [24]	USA	Prospective	127	FDG	Preoperative examinations, intraoperative histopathology and clinical findings	59	Low risk
7	Garcia-Barrecheguren et al. [10]	Spain	Prospective	24	FDG	Intraoperative results, histopathological and microbiological examinations	67.8	Low risk
8	Kiran et al. [20]	United Kingdom	Prospective	130	FDG	Histopathology and microbiological examinations	67.5	Low risk
9	Kobayashi et al. [35]	Japan	Prospective	65	Fluoride	Tissue examinations of surgically treated cases, serological and radiographic findings in conservatively treated cases	Not reported	Low risk
10	Kumar et al. [14]	India	Prospective	45	Fluoride	Intraoperative results, histopathological and microbiological examinations	54	Low risk
11	Kumar et al. [14]	India	Prospective	42	FDG	Intraoperative results, histopathological and microbiological examinations	53	Low risk
12	Kwee et al. [9]	Netherlands	Retrospective	78	FDG	Culture findings during the revision surgery	66.5	High risk
13	Love et al. [18]	USA	Retrospective	59	FDG	Intraoperative results, histopathological and microbiological examinations	Not reported	High risk
14	Manthey et al. [32]	Germany	Prospective	23	FDG	Culture findings following surgery	70	Low risk
15	Mayer-Wagner et al. [34]	Germany	Prospective	49	FDG	Culture findings following surgery	Not reported	Low risk
16	Mumme et al. [21]	Germany	Prospective	70	FDG	Intraoperative results, histopathological and microbiological examinations	68.7	Low risk
17	Pill et al. [15]	USA	Prospective	92	FDG	Clinical examination findings and preoperative, intraoperative findings	Not reported	Low risk
18	Reinartz et al. [28]	Germany	Prospective	92	Fluoride	Laboratory tests, radiological and clinical examination	68	Low risk
19	Stumpe et al. [23]	Switzerland	Prospective	35	FDG	Microbiological examination of the surgical specimens	64	Low risk
20	Teiler et al. [17]	Sweden	Prospective	10	FDG	Histopathological examinations	Not reported	Low risk
21	Tseng et al. [27]	Taiwan	Prospective	19	FDG	Intraoperative findings and microbiological analysis	64	Low risk
22	Vanquickenborne et al. [33]	Belgium	Prospective	17	FDG	Bacteriology of samples obtained by surgery or by needle aspiration and/or clinical findings	62	Low risk
23	Verberne et al. [28]	The Netherlands	Retrospective	33	FDG	Clinical examination findings and preoperative, intraoperative findings	76.4	High risk
24	Wang et al. [19]	China	Retrospective	103	Gallium	Preoperative and intraoperative serological results	61	High risk
25	Xu et al. [13]	China	Prospective	39	Gallium	Clinical, intraoperative results, histopathological and microbiological examinations	61.9	Low risk
26	Zhuang et al. [30]	USA	Prospective	38	FDG	Surgical exploration/clinical follow-up for one year	Not reported	High risk

*FDG* ^18^Fluorodeoxyglucose, *PET/CT* Positron Emission Tomography/Computed Tomography, *PJI* Prosthetic Joint Infection

### Diagnostic accuracy of PET/CT for periprosthetic joint infection

**Fig. 2:**
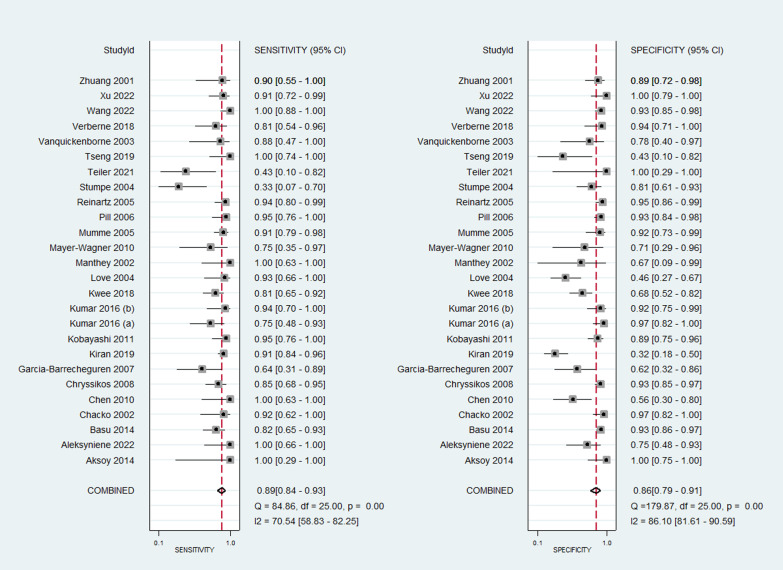
Forest plot showing the sensitivity and specificity of PET/CT for diagnosis of periprosthetic hip joint infection

**Fig. 3:**
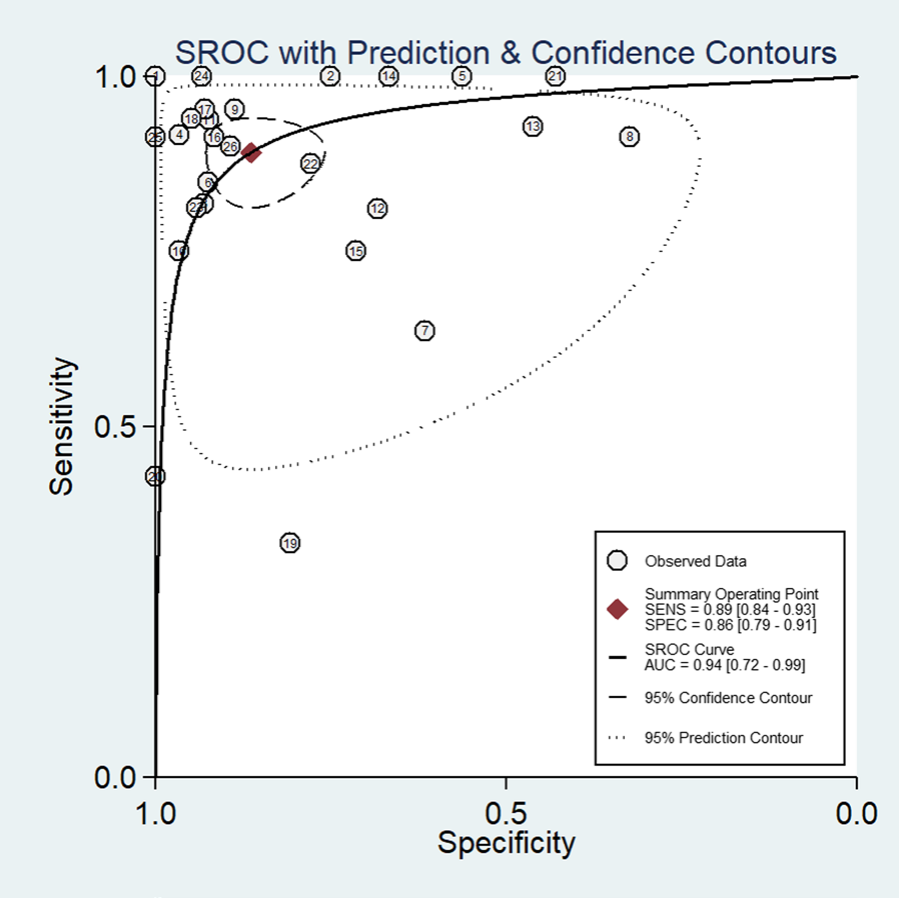
SROC Curve of PET/CT for diagnosis of periprosthetic hip joint infection

**Fig. 4:**
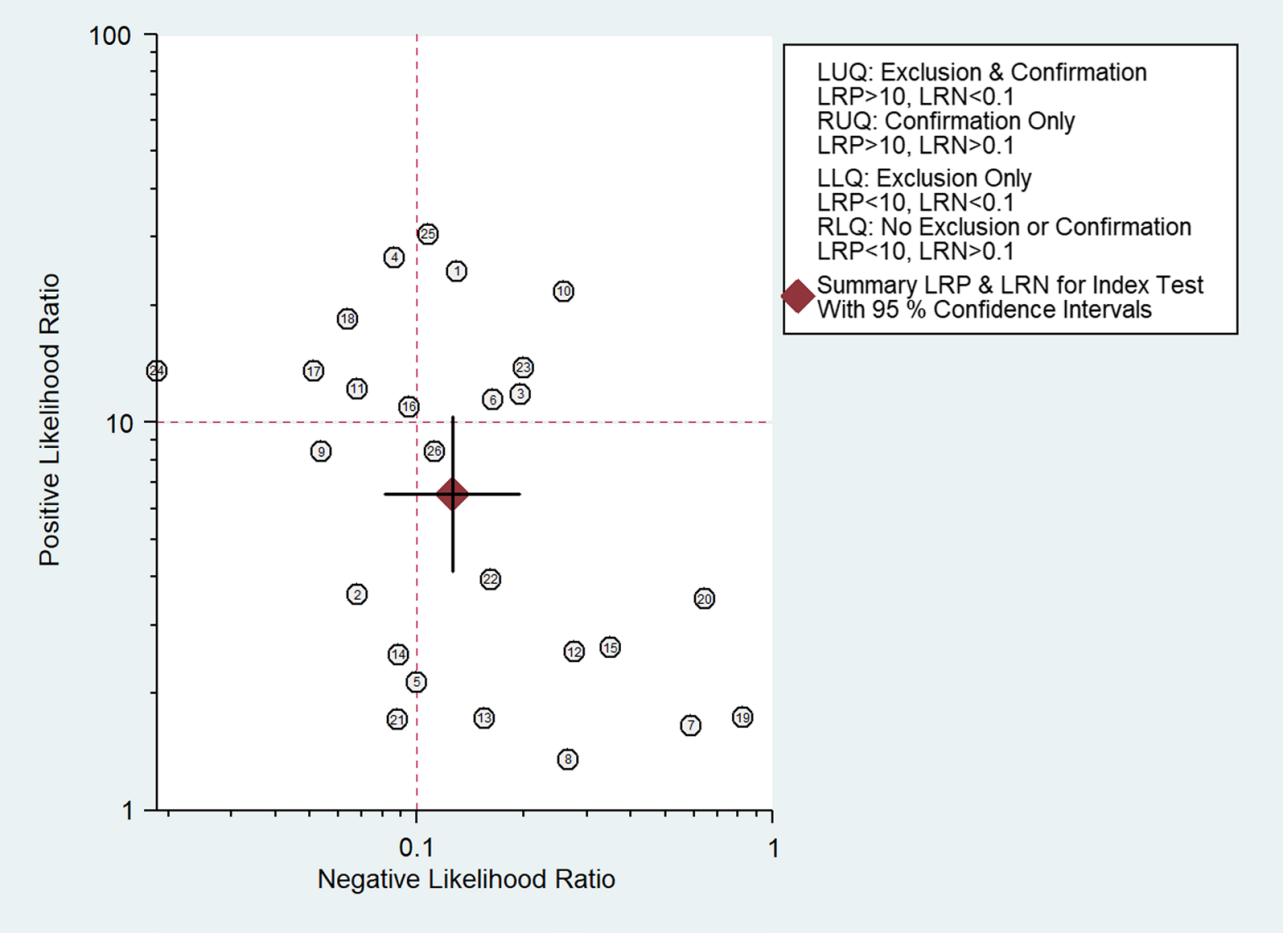
Likelihood scatter gram

The pooled sensitivity of PET/CT was 89% (95% CI 84–93%), while the pooled specificity was 86% (95% CI 79–91%) (Fig. 2). The AUROC was 0.94 (95% CI 0.72–0.99) (Fig. 3). There was statistically significant heterogeneity (*p* < 0.001) with I2 value of 96%. The diagnostic odds ratio was 52 (95% CI 26–106). Likelihood ratio positive was 6.5 (95% CI 4.1–10.3) and negative was 0.13 (95% CI 0.08–0.19). The likelihood ratio scattergram (Fig. 4) was generated to identify whether the imaging can be used for either confirmation or exclusion or both confirmation and exclusion. Both the likelihood ratios were placed in the right lower quadrant of the scattergram, indicating that the imaging cannot be used as a confirmatory test or to rule out the condition.

**Fig. 5:**
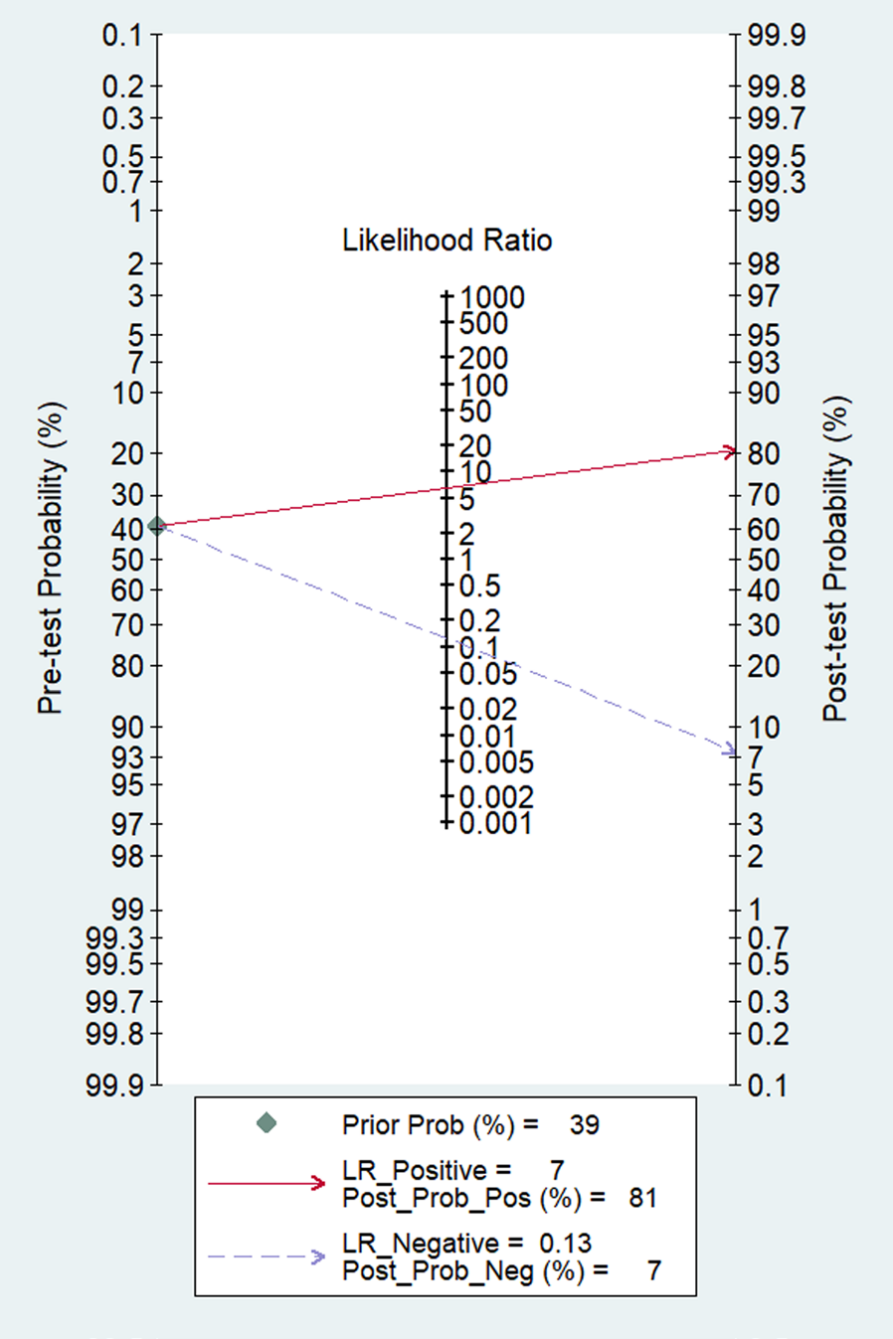
Fagan nomogram

**Fig. 6:**
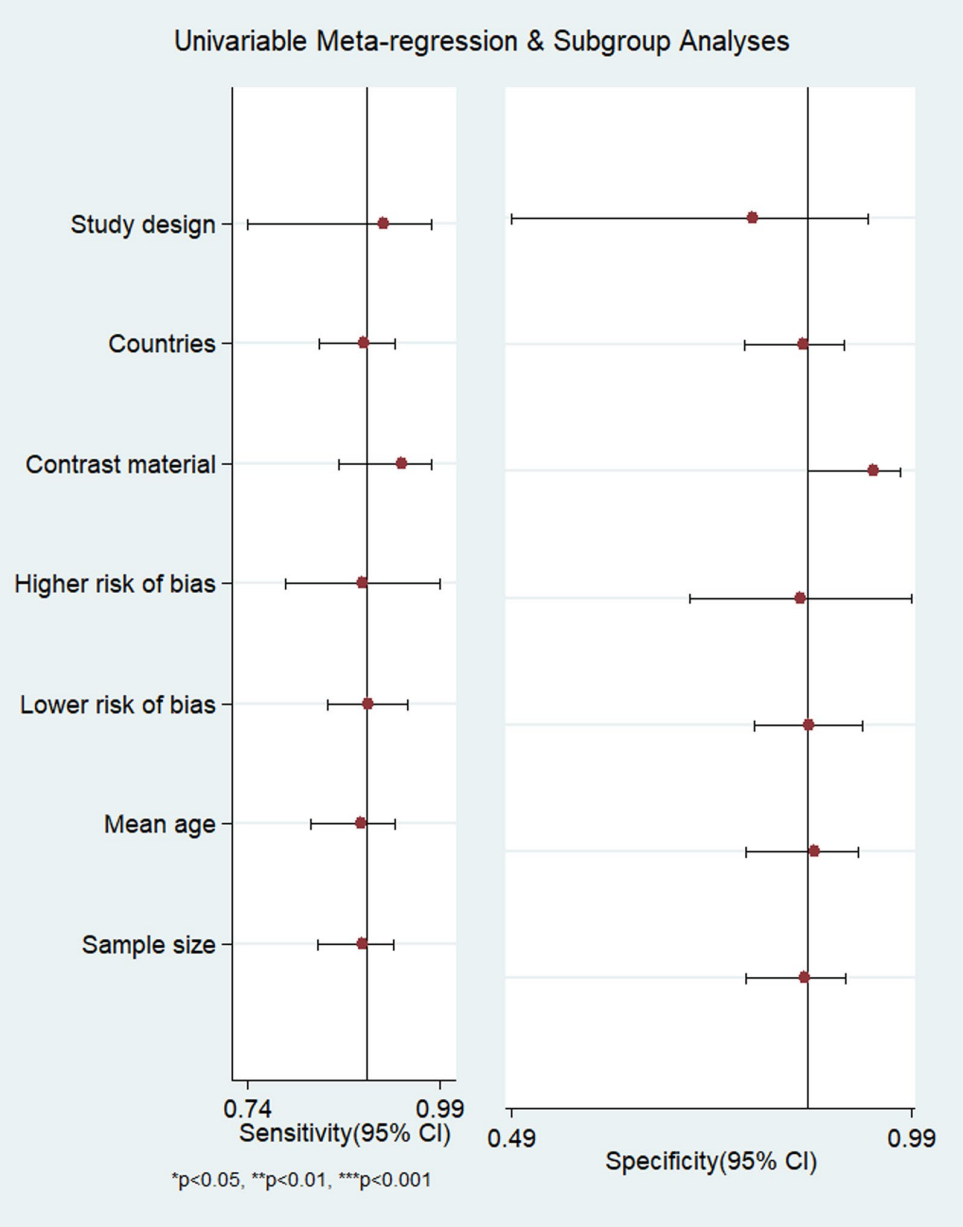
Meta-regression results

**Fig. 7:**
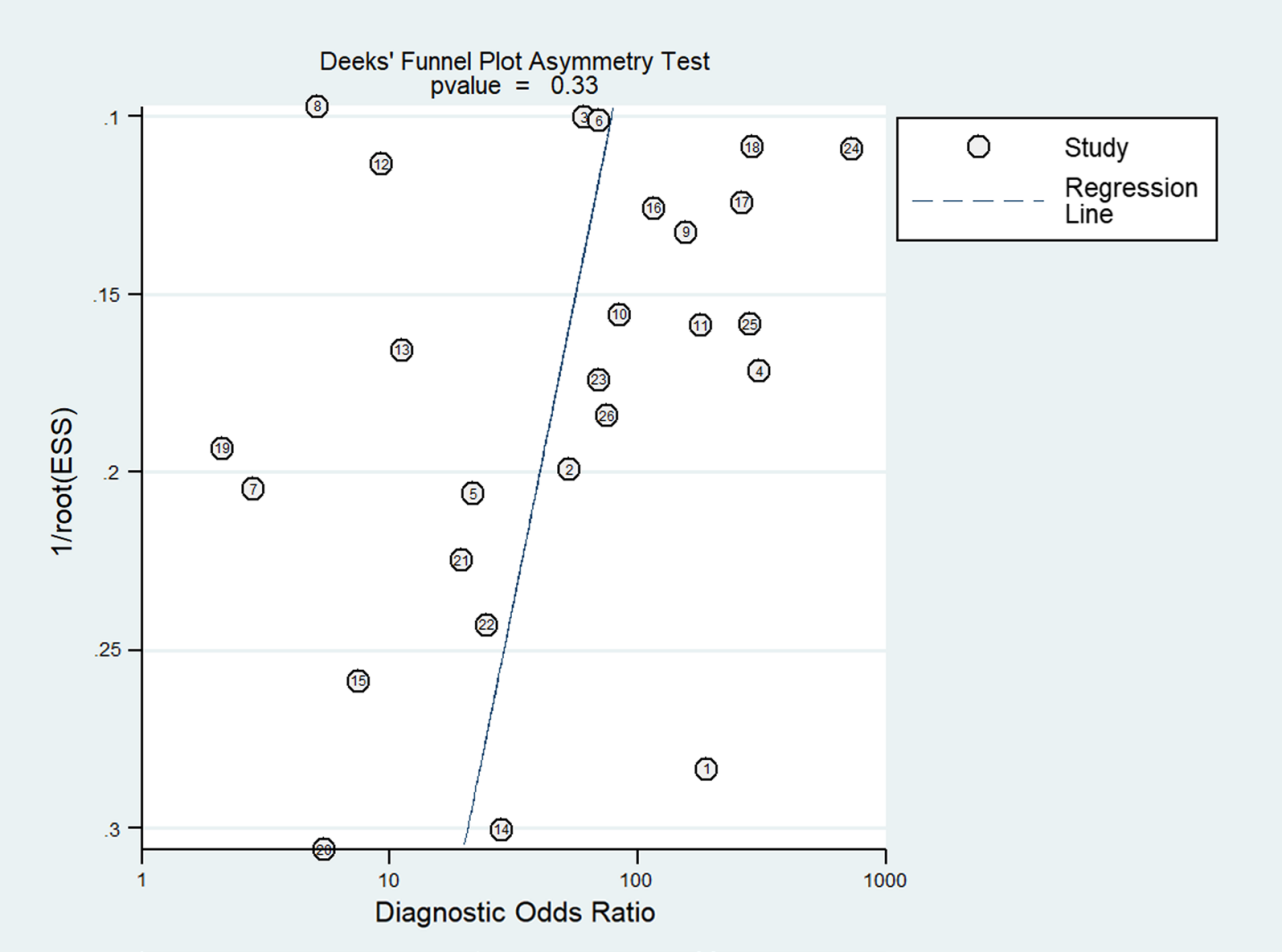
Funnel plot

Fagan nomogram (Fig. 5**)** was generated to identify the clinical application of the imaging technique. The nomograms showed very good clinical application with significant increase in the post-test probabilities compared to the pre-test probabilities. Meta-regression results did not find any factors significantly associated with the sensitivity or specificity model, but the type of contrast material and mean age showed significant association in the joint model (*p* < 0.001) (Fig. 6). Publication bias assessment showed a symmetrical plot with deek’s test showing a *p* value of 0.33 (Fig. 7).

**Fig. 8:**
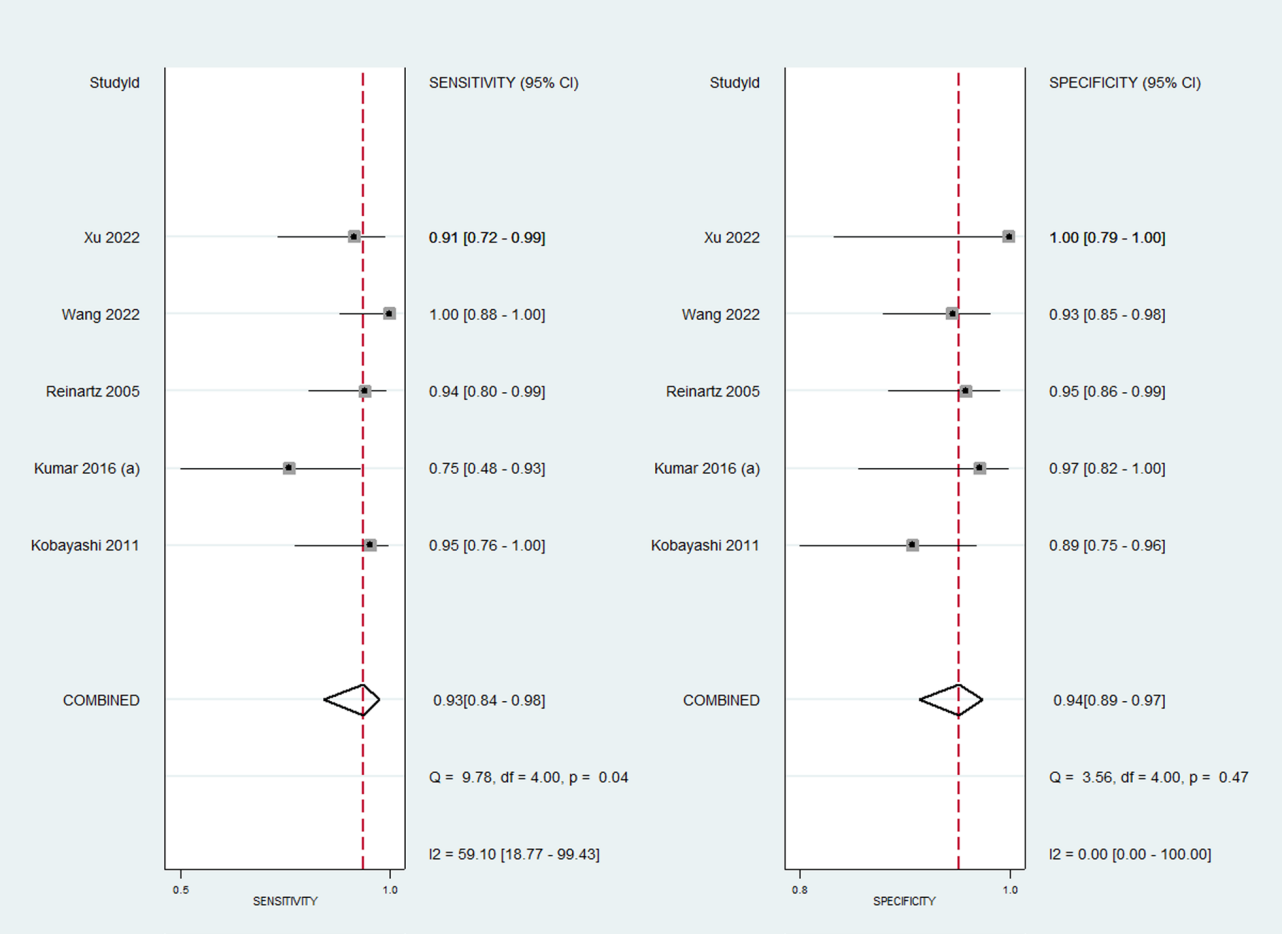
Forest plot showing the diagnostic accuracy of Fluoride/gallium PET scan

**Fig. 9:**
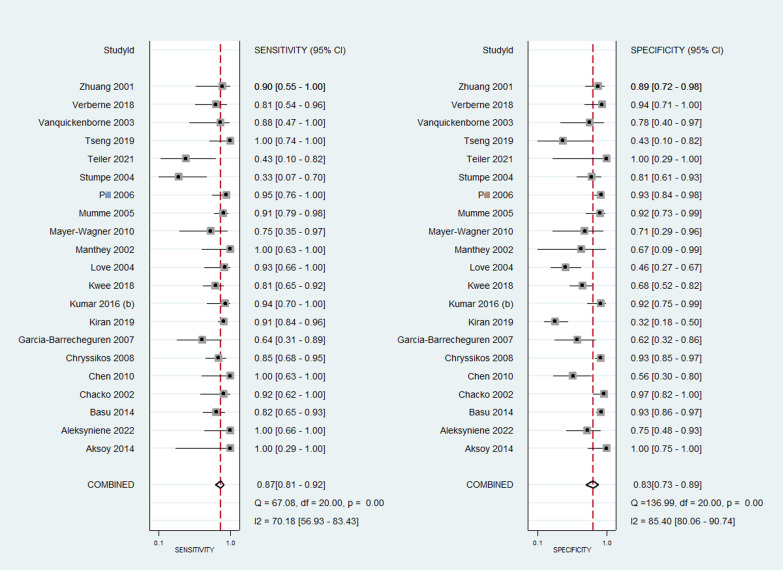
Forest plot showing the diagnostic accuracy of FDG-PET scan

Subgroup analysis based on the type of PET revealed that use of fluoride/gallium (Fig. 8) had higher sensitivity (93%; 95% CI 84–98%) and specificity (94%; 95% CI 89–97%) with AUC of 0.97 (95% CI 0.89–0.99) when compared to FDG-PET (sensitivity = 87%, 95% CI 81–92%; specificity = 83%, 95% CI 73–89%; AUC = 0.92; 95% CI 0.69–0.98) (Fig. 9).

## Discussion

PET/CT is a powerful imaging tool that can provide important information for the diagnosis and management of periprosthetic infections of the hip joint. PET/CT has several advantages over traditional imaging modalities such as X-ray and MRI in the evaluation of periprosthetic infections. Synthesizing the evidence showing its accuracy will help to inform the clinicians and decision makers to add the imaging technique to the guidelines and make it a standard protocol for these patients. Hence, this review was done to find the accuracy of PET/CT for diagnosing the periprosthetic infection of the hip.

In total, 26 of the identified studies were included in the review and analysis. Most studies were prospective and had lower risk of bias. PET/CT was found to have higher level of accuracy in terms of sensitivity (89%) and specificity (86%) with AUROC of 0.94. This finding was in line with the previous similar reviews on this topic [36–38]. However, the previous reviews have included studies irrespective of the site of infection and had limited number of studies when compared to the current review.

In addition to these general findings, our review further differentiated the accuracy based on the type of PET tracer used. The subgroup analysis revealed that the use of fluoride/gallium in PET/CT scanning yielded even higher accuracy than FDG-PET. Specifically, the sensitivity and specificity rates for fluoride/gallium were 93% (95% CI 84–98%) and 94% (95% CI 89–97%), respectively, with an AUC of 0.97 (95% CI 0.89–0.99). In comparison, FDG-PET demonstrated a slightly lower sensitivity of 87% (95% CI 81–92%) and specificity of 83% (95% CI 73–89%), with an AUC of 0.92 (95% CI 0.69–0.98). These results suggest that the choice of PET tracer can have significant implications for the diagnostic accuracy of PET/CT in the evaluation of periprosthetic infections of the hip joint. Particularly, the use of fluoride/gallium tracers seems to present a more effective option for diagnosing these infections, and it might be beneficial to update imaging protocols to preferentially use these tracers when available and appropriate.

The clinical application of PET/CT was also found to be appropriate, given the significant increase in the post-test probability in the nomogram compared to the pre-test probability. However, it did not satisfy the criteria to be used as either a confirmatory test or as a test to rule out the condition as per the LR scattergram. Nonetheless, the use of a radiotracer, such as 18F-fluorodeoxyglucose (18F-FDG), allows for the identification of increased metabolic activity, which is a characteristic of infected tissues [39]. This can be useful in detecting early or subtle signs of infection that may not be visible on other imaging modalities. Additionally, PET/CT provides functional and anatomic information in a single examination, which can be useful in the evaluation of complex cases where the distinction between infection and postoperative changes can be challenging [7]. By combining the functional information provided by the PET scan with the anatomic information provided by the CT scan, PET/CT scan aid in the localization and characterization of the infection.

Furthermore, PET/CT can be used to monitor the response to treatment, as it can detect resolution of the metabolic activity, which is an indication of the elimination of the infection. This can help in the management of periprosthetic infections of the hip joint by monitoring the treatment effectiveness. It is vital to highlight another recent clinical investigation which provides valuable context to our findings. This study conducted at the RWTH University Medical Centre of Aachen, Germany, revealed that the pathogens most frequently cultured were *S. epidermidis*, *S. aureus*, *E. faecalis*, and Methicillin-resistant *Staph aureus* (MRSA) in patients undergoing revision surgery for PJI of THA and TKA. Interestingly, the study reported that preoperative synovial fluid aspiration was positive in 37% of cases, intraoperative microbiology was positive in 85%, and bacteraemia was present in 17% of patients. These findings underscore the persistent challenge of PJIs, necessitating efficient diagnostic methods [40].

In summary, PET/CT can provide crucial information in the diagnosis and management of periprosthetic infections of the hip joint, due to its high sensitivity and specificity, its ability to provide functional and anatomic information in a single examination and its ability to monitor the treatment effectiveness [41].

The current study evidence has several strengths compared to previous reviews. First, this review contains the maximum number of studies on this research question. Second, most studies in our review were higher quality studies, enhancing the credibility of the available evidence. Third, there was no significant publication bias, further enhancing the reliability of the evidence. Finally, additional analysis to check the clinical utility, applicability and meta-regression was performed.

### Limitations

Our review also has certain limitations. There was significant heterogeneity in the study analysis, which was explored by means of meta-regression. Influence of various factors that might influence the accuracy parameters could not be explored, due to limitations of the study under each of the subcategories.

## Conclusions

Nonetheless, the current review findings are essential for the clinicians and surgeons involved in the management of patients with periprosthetic joint infection. PET/CT is a good enough diagnostic method and can be applied in patients suspected of having periprosthetic infection of the hip. To the already existing advantages in terms of time and cost reduction, the evidence of high level of accuracy can be added as another major advantage to be included in the standard protocol of these patients. Utilizing this imaging modality will help to start the treatment early leading to better patient outcomes. More prospective studies can be performed to check whether this can be used as confirmatory test, as it has not reached such levels as per the current review findings. Further large-scale research can help to find answers for such questions and provide final conclusive evidence on the inclusion of the imaging modality into the routine clinical practice guidelines for suspected periprosthetic joint infection patients.

### Supplementary Information

**Additional file 1.** Search Strategy.

## Data Availability

The datasets used and/or analysed during the current study are available from the corresponding author on reasonable request.
