# Correction: Diagnostic accuracy of positron emission tomography/computerized tomography for periprosthetic joint infection of hip: systematic review and meta-analysis

**DOI:** 10.1186/s13018-023-04345-9

**Published:** 2023-11-14

**Authors:** Hongning Hua, Jinwen Liu

**Affiliations:** 1 Department of Emergency Surgery, Shanxi Bethune Hospital, Shanxi Academy of Medical SciencesTongji Shanxi Hospital, Third Hospital of Shanxi Medical University, Taiyuan, 030032 China; 2 Department of Emergency Surgery, Tongji Hospital, Tongji Medical College, Huazhong University of Science and Technology, Wuhan, 430030 China; 3 Interventional Treatment Room, Shanxi Bethune Hospital, Shanxi Academy of Medical Sciences, Tongji Shanxi Hospital, Third Hospital of Shanxi Medical University, Taiyuan, 030032 China; 4 Interventional Treatment Room, Tongji Hospital, Tongji Medical College, Huazhong University of Science and Technology, Wuhan, 430030 China

**Correction: Journal of Orthopaedic Surgery and Research (2023) 18:640** **https://doi.org/10.1186/s13018-023-04061-4**

Following publication of the original article [1], the author has identified an error in the affiliation of the authors. The corrected affiliations of the authors are given below.

The authors apologise for this error”.
