# Supplementary material for: Diagnostic accuracy of positron emission tomography/computerized tomography for periprosthetic joint infection of hip: systematic review and meta-analysis
Source: J Orthop Surg Res. 2023 Aug 30;18:640. doi: 10.1186/s13018-023-04061-4 (PMC10466775; doi:10.1186/s13018-023-04061-4)
Supplement: Supplementary file 1 — Additional file 1. Search Strategy. [file 13018_2023_4061_MOESM1_ESM.pdf]

Search Strategy:

("positron emission tomography computed tomography"[MeSH Terms] OR ("positron"[All Fields] AND "emission"[All Fields] AND "tomography"[All Fields] AND "computed"[All Fields] AND "tomography"[All Fields]) OR "positron emission tomography computed tomography"[All Fields] OR ("pet"[All Fields] AND "ct"[All Fields]) OR "pet ct"[All Fields]) AND ("joint s"[All Fields] OR "joints"[MeSH Terms] OR "joints"[All Fields] OR "joint"[All Fields]) AND ("infect"[All Fields] OR "infectability"[All Fields] OR "infectable"[All Fields] OR "infectant"[All Fields] OR "infectants"[All Fields] OR "infected"[All Fields] OR "infecteds"[All Fields] OR "infectibility"[All Fields] OR "infectible"[All Fields] OR "infecting"[All Fields] OR "infection s"[All Fields] OR "infections"[MeSH Terms] OR "infections"[All Fields] OR "infection"[All Fields] OR "infective"[All Fields] OR "infectiveness"[All Fields] OR "infectives"[All Fields] OR "infectivities"[All Fields] OR "infects"[All Fields] OR "pathogenicity"[MeSH Subheading] OR "pathogenicity"[All Fields] OR "infectivity"[All Fields])

Translations

PET/CT: "positron emission tomography computed tomography"[MeSH Terms] OR ("positron"[All Fields] AND "emission"[All Fields] AND "tomography"[All Fields] AND "computed"[All Fields] AND "tomography"[All Fields]) OR "positron emission tomography computed tomography"[All Fields] OR ("pet"[All Fields] AND "ct"[All Fields]) OR "pet ct"[All Fields]

Joint: "joint's"[All Fields] OR "joints"[MeSH Terms] OR "joints"[All Fields] OR "joint"[All Fields]

Infection: "infect"[All Fields] OR "infectability"[All Fields] OR "infectable"[All Fields] OR "infectant"[All Fields] OR "infectants"[All Fields] OR "infected"[All Fields] OR

"infecteds"[All Fields] OR "infectibility"[All Fields] OR "infectible"[All Fields] OR "infecting"[All Fields] OR "infection's"[All Fields] OR "infections"[MeSH Terms] OR "infections"[All Fields] OR "infection"[All Fields] OR "infective"[All Fields] OR "infectiveness"[All Fields] OR "infectives"[All Fields] OR "infectivities"[All Fields] OR "infects"[All Fields] OR "pathogenicity"[Subheading] OR "pathogenicity"[All Fields] OR "infectivity"[All Fields] **– 173 results**
